# Beliefs and Information Seeking in Patients With Cancer in Southwest China: Survey Study

**DOI:** 10.2196/16138

**Published:** 2020-08-21

**Authors:** Juan Xie, Shi Xie, Ying Cheng, Zhe He

**Affiliations:** 1 School of Information Management Nanjing University Nanjing China; 2 Jinling College Nanjing University Nanjing China; 3 School of Information College of Communication and Information Florida State University Tallahassee, FL United States

**Keywords:** cancer information seeking, cancer belief, fatalism, southwest China

## Abstract

**Background:**

Although previous studies have reported the cancer information-seeking behaviors among patients in high-income countries, the cancer information-seeking practices of patients living in low- and middle-income areas are less known.

**Objective:**

This study investigated the beliefs and information-seeking patterns of cancer patients in southwest China.

**Methods:**

A questionnaire was designed, and data were collected in two hospitals (N=285) in southwest China. Statistical analyses included bivariate analyses and regressions.

**Results:**

Patients’ attitudes towards cancer fatalism were significantly influenced by marital status (*P*<.001), education (*P*<.001), and household income (*P*<.001). Moreover, endorsing fatalistic belief was positively associated with age (r=0.35, *P*<.001). The regression model showed that younger patients (odds ratio [OR] 0.96, 95% CI 0.93-0.99) and those with higher education (OR 1.75, 95% CI 1.09-2.81) were more likely to seek information. Additionally, patients who were less confident in getting information were more likely to find information (OR 1.70, 95% CI 1.15-2.52), while fatalism belief was not significant in the regression (OR 0.65, 95% CI 0.22-1.95).

**Conclusions:**

This study explored the information-seeking patterns of cancer patients in southwest China. It was found that many Chinese people endorsed cancer fatalism. These pessimistic beliefs about the potential to prevent and to cure cancer correlate with rather than cause cancer-related information seeking. However, self-efficacy about the confidence in finding needed cancer information was a significant predictor of information-seeking.

## Introduction

### Background

According to the estimates of World Health Organization, cancer is the second leading cause of global death and is now responsible for 1 in 6 deaths [1]. Information about cancer can benefit patients in decision making, coping with treatment, their psychological well-being, and their quality of life [2,3]. Studying the patterns of patients’ cancer-related information-seeking is thus crucial to high-quality provider-patient communication, improved cancer care, and improved outcomes.

Although previous studies have reported information seeking among cancer patients in high-income countries such as the United States and some European countries [4,5], little is known about cancer-related information patterns among patients living in low- and middle-income regions, where approximately 70% of deaths from cancer occur [1]. In China, while increasing attention has been paid to cancer control and cancer information dissemination [6], most of the work has been conducted in eastern metropolitan regions such as Beijing [7], Hefei [7], and Nanjing [8], leaving cancer patients in the southwest underrepresented. Southwest China consists of 5 provinces and is 14.40% of China’s total population [9]. By 2017, the GDP (gross domestic product) of this region only accounted for 10% of the total GDP [9]; yet, according to national cancer statistics, southwest China had the highest cancer incidence rates, highest cancer mortality rates, and the lowest cancer survival rates in the country [10]. Possible reasons for these include a higher smoking prevalence, limited medical resources, and inadequate cancer screening and treatment [10]. Thus, the education and cancer care of patients in southwest China are concerning.

People’s attitudes and beliefs can influence their intentions and behaviors [11]. For example, beliefs about cancer were associated with help-seeking behavior [12]. Furthermore, it was found that Asian individuals were more likely to have fatalistic cancer beliefs [13], for example, that the outcome of cancer is predetermined and arranged by fortune and predestination, and that nothing can be done to control or change the outcome of cancer [14]. One study [15] explored the role of self-efficacy and found that higher self-efficacy beliefs were positively correlated with cancer-screening intentions. However, most of these studies focused on the general public, while the beliefs among cancer patients, especially those from underrepresented regions, are less known.

### Prior Work

#### Cancer Patients’ Information Seeking

A seminal work [16] identified the individual differences in cancer information exchange as monitoring and blunting. *Monitoring* indicated the active seeking of information related to cancer, while *blunting* indicated the avoidance of threatening information. Later studies [17-19] further categorized active information behavior as information seeking and information scanning, arguing that scanning was a less active behavior that gathered and came across relevant information incidentally. Information scanning often occurred through mass media [20]. Moreover, Lambert, et al [21] delineated patients’ cancer-related information-seeking preferences through an in-depth qualitative study, including three types of active search (intense, complementary, and fortuitous) and two types of information avoidance (minimal and guarded). These five patterns differed in essential characteristics such as the type and amount of information needs, as well as sources. A recent study [2] showed that most people were active seekers, although the prevalence of information avoidance was higher than expected.

Based on theoretical frameworks such as the Health Belief Model [22] and the Comprehensive Model of Information Seeking [23], previous studies [19,24-26] have found differences between information seekers and nonseekers among cancer patients. These studies investigated demographic characteristics (eg, gender, age, income, education) first, and then explored the effects of social determinants (such as having a regular health care provider, salience, cancer type, cancer stage, and treatment type); however, among the different studies, the effects of cancer type and cancer stage were often found to be inconsistent.

Comprehensive reviews [27,28] of information need and sources of information among cancer patients have been conducted; the majority of previous studies focused on patients in diagnosis and treatment stages. Therefore, the most prevalent information need was treatment-related, such as a need for information on the side effects of treatments and treatment options [4,28]. Furthermore, the most frequently used information source was health professionals, followed by cancer survivors and the internet [4,24,29]. Additionally, the differences for information need and sources used between patients of different age, gender, ethnicity or cancer type have also been explored [19,30,31]. For example, a meta-analysis [30] showed that younger cancer patients need more information.

#### Cancer Beliefs

An individual’s beliefs about cancer can affect the way that person copes with cancer-related issues. People holding fatalistic or negative beliefs were found to have delayed diagnoses, avoid screening, and have worse survival outcomes [32]. Cancer fatalism has been operationalized by pessimism, helplessness, and confusion about how to prevent cancer. For example, based on the Health Information National Trends Survey (HINTS) [33], Kobayashi and Smith [34] found that a common perception in the US population was that everything caused cancer, one-third did not believe cancer was preventable, and more than half automatically associated cancer with death. Moreover, some research [13,32,35,36] has suggested ethnic and cultural differences in fatalism about cancer prevention, arguing that people from Eastern cultures tend to hold a more fatalistic attitude; they were found to be less likely to engage in cancer-related information-seeking, cancer screening, and cancer-preventive behaviors such as exercising and having a healthy diet. Cancer-related information seeking was also found to have an impact on the beliefs. For example, Lee et al [37] found that among those with a lower education level and less health knowledge, health-related internet use reduced cancer fatalism.

Previous studies [38] related to cancer beliefs focused mainly on the general public. However, little is known about the beliefs of cancer patients or the relationship between patients’ beliefs and their health information seeking. A Turkish study [39] found that the majority of cancer patients and half of their relatives considered cancer as curable and preventable, and their beliefs influenced their wish for information and their efforts to obtain it. To improve the quality of the cancer care continuum, more research about cancer beliefs among patients during treatment and survival is necessary.

### Goal of This Study

This study aimed to examine the cancer belief and self-efficacy of cancer patients in southwest China, and the factors influencing their cancer information seeking. A survey was conducted in two hospitals in Chengdu and Meishan. Statistical analyses included bivariate analyses and regression. Findings from this study can help develop effective cancer communication interventions and education strategies to achieve high-quality cancer care.

## Methods

### Overview

A survey was conducted to explore the cancer beliefs and information seeking of cancer patients in southwest China. The variables of the survey were measured with a self-report questionnaire (Multimedia Appendix 1) which included demographic questions and questions about cancer belief, self-efficacy, and cancer information seeking. The survey was conducted between January 2019 and April 2019.

### Recruitment

Patients with cancer were recruited by convenience sampling in Meishan Tumor Hospital and the oncology inpatient department of the First Affiliated Hospital of Chengdu Medical College, located in Meishan and Chengdu, respectively, in southwest China. The two hospitals vary in levels: Chengdu Medical College is a level 3 hospital and Meishan Tumor Hospital is a level 2 hospital. Eligibility criteria for participation were patients who were diagnosed with cancer and who were undergoing treatment. We did not select patients by cancer type or gender because of ethical considerations required by the hospital administrations. One researcher together with a doctor distributed the questionnaires during the doctor’s rounds. Each patient was invited to read and sign an informed consent form, which explained the aims, demands, guaranteed anonymity of the study, and that refusing to participate would not influence treatment. They were also asked to answer the questions carefully and were told that the researcher was available when they needed help.

With the aim of estimating the pattern of patients seeking cancer information in southwest China, we calculated the sample size by referring to a sample size calculation for qualitative variable in cross-sectional studies [40]. A previous study [7] in China showed that the portion of participants who reported having looked for information related to cancer may not be more than 28%, so the target sample size for the current study was determined to be 310. Three hundred questionnaires were distributed.

### Measures

#### Cancer Belief

Cancer belief was assessed according to the HINTS (Health Information National Trends Survey) [41] through a guide question “How much do you agree or disagree with each of the following statements?” followed by 5 items: (1) “It seems like everything causes cancer,” (2) “There’s not much you can do to lower your chances of getting cancer,” (3) “There are so many different recommendations about preventing cancer, it's hard to know which ones to follow,” (4) “In adults, cancer is more common than heart disease,” (5) “When I think about cancer, I automatically think about death.” Responses for each item were rated using a 5-point Likert scale ranging from “strongly disagree” to “strongly agree.” The internal consistency of these 5 items was (Cronbach α=.75). The measurement consisting of 5 items was more reliable (Cronbach α>.70) than the measurement with 3 items which has frequently been used in previous studies [34]; therefore, we used the mean of the 5 items to create a fatalism scale. Items adapted from HINTS5 were translated into Mandarin through back translation and group discussion to guarantee linguistic equivalence between the English and Chinese versions.

#### Self-Efficacy

Self-efficacy suggests how confident patients are in getting health advice or information they need. From the HINTS [41], we used one item “I am NOT confident in finding cancer-related information I need.” The response was rated using a 5-point Likert scale rating ranging from “strongly disagree” to “strongly agree.”

#### Demographic Variables

Based on previous studies [34,37,42] related to cancer information seeking, and the different information practices among cancer patients with different demographic characteristics, we controlled for demographic variables. These variables included age, marital status (single, never been married; married; widowed; divorced), education (primary school or lower, junior school, high school, some college, bachelor’s degree or higher), and household annual income (less than 10,000 RMB or approximately US $1425; 10,000 to 50,000 RMB; 50,000 to 100,000 RMB; greater than 100,000 RMB). As required by the hospital administrations, we did not collect information on participants’ gender and their cancer types to avoid identification.

#### Dependent Variable

Cancer information seeking was measured with 1 item “Have you ever looked for information about cancer from any source?” The response was “yes” or “no.” Participants who answered “no” were considered nonseekers.

### Statistical Analysis

To investigate the relationship between patients’ beliefs and their cancer-related information-seeking behavior, we conducted bivariate analyses as well as multivariate regression analysis. First, the essential characteristics among different group of participants were described. Subsequently, the beliefs and information-seeking differences in demographic variables except age were examined using an analysis of variance (ANOVA) and cross-tabulation (Pearson chi-square test). The relationships between age and beliefs were tested using Pearson correlation while the differences between information seekers and nonseekers were analyzed using two-tailed independent *t* tests. Moreover, the self-efficacy and cancer belief differences between information seekers and nonseekers were explored using two-tailed independent *t* tests. Finally, variables were fitted in a logistic regression to investigate their predictive relationship with cancer information-seeking behavior, before which collinearity diagnostics were conducted (Multimedia Appendix 2). The categorical variable (marital status) was transformed into a dummy variable in the regression. All the analyses were conducted in SPSS (version 23.0; IBM Corp) and the significance level was *P*<.05.

## Results

### Descriptive Statistics

Demographic characteristics of participants (N=285) are shown in Table 1. Among the 285 patients who completed the survey, the mean age was 51.79 (range 17-95) years old. Most were married (206/285, 72.3%), while the number of patients in the other three categories of marital status were close; and 25.6% (73/285) reported a junior school level of education, followed by high school, primary school or lower, college, and bachelor’s degree or higher. Moreover, 48.4% (138/285) reported a household annual income lower than 10,000 RMB, while the distribution of patients in the other three income categories was fairly equivalent.

The majority of patients (233/285, 81.8%) had looked for cancer-related information. Cancer belief (mean 3.23, SD 0.52) showed a moderate or strong fatalistic view. Among the 5 types of fatalism, “inevitable death” was most common (mean 3.66, SD 0.95), followed by “helplessness” (mean 3.18, SD 0.75), and “prevalence” (mean 3.17, SD 0.70). Furthermore, confidence in getting cancer information among 108 patients was moderate (108/285, 37.9%), while 6.0% (17/285) strongly acknowledged their confidence.

**Table 1:** Participant demographic data.

Characteristics (N=285)		Patients, n (%)
**Age**		
	≤19	6 (2.1)
	20-29	23 (8.1)
	30-39	43 (15.1)
	40-49	72 (25.3)
	50-59	50 (17.5)
	60-69	36 (12.6)
	70-79	31 (10.9)
	80-89	20 (7.0)
	≥90	4 (1.4)
**Marital status**		
	Single	26 (9.1)
	Married	206 (72.3)
	Widowed	30 (10.5)
	Divorced	23(8.1)
**Education**		
	Primary school or lower	66 (23.2)
	Junior school	73 (25.6)
	High school	67 (23.5)
	Some college	45 (15.8)
	Bachelor’s degree or higher	34 (11.9)
**Household income (in RMB^a^)**		
	<10,000	138 (48.4)
	10,000-50,000	58 (20.4)
	50,000-100,000	42 (14.7)
	>100,000	47 (16.5)

^a^At the time of publication, an exchange rate of approximately US $1=0.19 RMB was applicable.

### Beliefs and Demographics

We conducted bivariate analyses to investigate the relationship between patients’ demographics and their beliefs. Patients’ attitudes towards cancer fatalism were significantly related to marital status (*P*<.001), education (*P*<.001), and household income (*P*<.001) (Table 2). Moreover, endorsing fatalistic belief was moderately associated with age (r=0.35, *P*<.001). A linear regression analysis was conducted to further test the relationship between demographics and cancer fatalism (Multimedia Appendix 3). It was found that age (*P*=.003), education (*P*<.001), and household income (*P*=.04) still showed significant correlation with cancer fatalism. On the other hand, marital status (*P*<.001), education (*P*<.001), and household income (*P*<.001) were also related to patients’ self-efficacy. Furthermore, older patients were more likely to believe in their ability to obtain the information they need (r=–0.41, *P*<.001). These results were also tested in a linear regression model, in which age (*P*<.001) and education (*P<*.001) remained significant (Multimedia Appendix 3).

**Table 2:** Results of bivariate analyses.

Variables		Cancer belief		Self-efficacy	
		Value	*P* value	Value	*P* value
**Marital status, mean (SD)**			<.001^a^		<.001^a^
	Single	2.77 (0.44)		4.08 (0.89)	
	Married	3.23 (0.53)		2.88 (1.05)	
	Widowed	3.47 (0.28)		2.53 (0.97)	
	Divorced	3.44 (0.52)		2.44 (1.31)	
**Education, mean (SD)**			<.001^a^		<.001^a^
	Primary school or lower	3.74 (0.37)		2.38 (0.82)	
	Junior school	3.40 (0.34)		2.47 (0.97)	
	High school	3.11 (0.39)		2.96 (1.12)	
	Some college	2.88 (0.35)		3.44 (1.10)	
	Bachelor’s degree or higher	2.69 (0.40)		4.15 (0.82)	
**Household annual income, mean (SD)**			<.001^a^		<.001^a^
	<10,000 RMB	3.27 (0.51)		2.88 (0.99)	
	10,000-50,000 RMB	3.28 (0.51)		2.59 (1.09)	
	50,000-100,000 RMB	3.39 (0.44)		2.67 (1.12)	
	>100,000 RMB	2.92 (0.53)		3.64 (1.19)	
Age, r		0.35	<.001^b^	–0.41	<.001^b^

^a^ANOVA.

^b^Pearson correlation.

### Cancer Information Seeking and Demographics

Our results suggested significant differences between cancer information seekers and nonseekers in marital status (*P*<.001) and education (*P*<.001), while household annual income showed no influence (*P*=.45) (Table 3), and the mean age of nonseekers was significantly higher than that of seekers (*P*<.001). However, the effect of income became insignificant in the logistic regression (odds ratio [OR] 1.01, 95% CI 0.68-1.48) (Table 4). In the regression model, education level and age were demographic predictors of cancer information-seeking behavior, with younger patients (OR 0.96, 95% CI 0.93-0.99) and those with higher education (OR 1.75, 95% CI 1.09-2.81) being more likely to look for information.

**Table 3:** Comparison between patients who seek information and those who do not.

Variables		Cancer information seeking		
		Seekers (n=233)	Nonseekers (n=52)	*P* value
**Marital status, n (%)**				<.001^a^
	Single	26 (11.2)	0 (0)	
	Married	173 (74.2)	33 (63.5)	
	Widowed	15 (6.4)	15 (28.8)	
	Divorced	19 (8.1)	4 (7.8)	
**Education, n (%)**				<.001^a^
	Primary school or lower	39 (16.7)	27 (51.9)	
	Junior school	58 (24.9)	15 (28.8)	
	High school	61 (26.2)	6 (11.5)	
	Some college	42 (18.0)	3 (5.8)	
	Bachelor’s degree or higher	33 (14.2)	1 (1.9)	
**Household annual income, n (%)**				.45^a^
	<10,000 RMB	109 (46.8)	29 (55.8)	
	10,000-50,000 RMB	47 (20.2)	11 (21.2)	
	50,000-100,000 RMB	35 (15.0)	7 (13.5)	
	>100,000 RMB	42 (18.0)	5 (9.6)	
Age, mean		48.70 (15.97)	65.67 (16.91)	<.001^b^
Cancer belief, mean (SD)		3.16 (0.52)	3.56 (0.37)	<.001^b^
Self-efficacy, mean (SD)		3.09 (1.04)	2.14 (1.14)	<.001^b^

^a^Pearson chi-square test.

^b^*t* test.

**Table 4:** Results of logistic regression.

Variables^a^	B	SE	*P* value	OR^b^ (95% CI)
Education	0.56	0.24	.02	1.75 (1.09, 2.81)
Household annual income	0.01	0.20	.98	1.01 (0.68, 1.48)
Marital_single^c^	16.50	7451.17	.99	14576924.17 (0,—)
Marital_married	–0.12	0.65	.85	0.89 (0.25, 3.18)
Marital_widowed	–0.85	0.82	.30	0.43 (0.09, 2.15)
Age	–0.042	0.01	.004	0.96 (0.93, 0.99)
Cancer belief	–0.42	0.56	.45	0.65 (0.22, 1.95)
Self-efficacy	0.53	0.20	.009	1.70 (1.14, 2.52)
Constant	2.95	2.20	.18	19.14 (0,—)

^a^Model summaries (Cox and Snell R^2^= 0.24; Nagelkerke R^2^=0.38).

^b^OR: odds ratio.

^c^This implausibly large odds ratio value was possibly due to the uneven distribution among marital groups, and it should be interpreted with caution.

### Cancer Information Seeking and Beliefs

Patients’ attitudes towards cancer fatalism (*P*<.001) and their self-efficacy (*P*<.001) were significantly associated with whether to seek information or not (Table 3). Cancer fatalism was higher in the nonseeking group (nonseeking: mean 3.56; seeking: mean 3.16) and this group also showed a higher level of confidence (nonseeking: mean 2.14; seeking: mean 3.09; it is notable that in the scale of self-efficacy, a higher value means a greater possibility of agreement that the participant is not confident). However, when tested in the regression model, cancer belief was not found to be a predictor for information seeking (OR 0.65, 95% CI 0.22-1.95) (Table 3), suggesting a complicated relationship between these two variables. Additionally, patients who were less confident in getting information were more likely to find information (OR 1.70, 95% CI 1.15-2.52). 

## Discussion

### Principal Results

To the best of our knowledge, this is the first study to investigate the beliefs and information-seeking behavior of cancer patients in southwest China. It was found that many endorsed cancer fatalism. These pessimistic beliefs about the potential to prevent and to cure cancer were correlated with rather than the cause of cancer-related information seeking. However, self-efficacy was a significant predictor of seeking out information (*P*<.001). Besides, it was found that patients who were younger (*P*<.001) and with higher level of education (*P*<.001) were more likely to find information. The response rate was quite high (285/300, 95%), which might because (1) we surveyed hospitalized patients, and (2) they were familiar with their doctors and were thus willing to answer the questions.

### The Influencing Factors of Cancer Information Seeking

Cancer fatalism is a multidimensional concept often operationalized by beliefs of pessimism, fear, helplessness, confusion, and inevitable death [39]. A previous study [13] found that people from Asian cultures tend to hold a more fatalistic view on negative issues such as having cancer, considering them to be unpreventable and out of one’s control. Moreover, the same results have been reported among Asian communities in Western countries [35]; in these communities, individuals were more likely to associate cancer with bad luck, punishment for sins committed in the current or previous life, or the will of a supreme being [43], embedded in religious traditions such as Taoism. The higher fatalism among cancer patients in Chinese society was supported in our study (mean 3.23, SD 0.52). In addition, a study [7] found that the average fatalistic attitude of Chinese public was also slightly above the scale midpoint (mean 3.30, SD 0.80, range 1-5). The similar results suggest that cancer care intervention in our study’s region failed to dispel fatalistic views. Additionally, in this study, we found that older patients were more likely to endorse fatalistic beliefs, while those who were better educated held more positive perceptions about cancer, as supported by a Turkish research [39].

Although a previous study [36] found that people who held a negative cancer belief were more likely to be information avoiders; our results did not support this: in the logistics model, there was no predictive relationship between cancer belief and information seeking. One possible explanation is that they might influence each other—on one hand, in the literature, it has been reported that greater information seeking led to decreased fatalism [44], internet-use reduced fatalism [37], and negative health information–seeking experiences might contribute to cancer fatalism [45]; on the other hand, some studies [7,46] found that fatalistic attitudes about cancer were an important barrier to information seeking. A significant correlation was found in our bivariate analysis (*P*<.001), but cancer fatalism was not a predictor in the regression model (*P*=.45; OR 0.65, 95% CI 0.22-1.95). The bidirectional effects of cancer fatalism and information seeking could be further investigated through a qualitative approach.

In this study, we investigated how confident patients were of their ability to find cancer-related information. Interestingly, we found that older patients were more confident, while those who had higher incomes and were more educated reported to be less confident. One theoretical explanation could be optimistic beliefs about cancer risk among Asians [13]. This belief system allows them to underestimate their chance of suffering from serious outcomes [47]. However, patients with higher socioeconomic status were more likely to be exposed to a more westernized lifestyle and worldview, making them more self-enhancing and self-critical. Thus, older people were found to be more confident than those with higher income and education. On the other hand, as shown in the regression model, patients who were less confident about their information-seeking ability were more likely to look for information. This finding is inconsistent with studies [48,49] that have found that efficacy encourages health information–seeking. The reasons may be two-fold: first, we did not measure self-efficacy through a standard scale as the previous studies did; second, patients reporting less confidence tended to be younger, more educated, and were more likely to seek information. Therefore, the effect of self-efficacy on information seeking deserves future research. Additionally, younger and more educated patients might expect more from the health care system and keep on seeking, since a study found that intense seekers were more likely to be dissatisfied with the cancer information provided [2].

A large percentage of our respondents reported a low household income and low education level, which suggests social and economic gaps between the eastern and western China. For example, in two eastern cities (Beijing and Hefei, Anhui province), the majority of households earn 60,001 RMB or more annually [7], while most participants in this study had an annual household income of 10,000 RMB or lower.

Our analyses also revealed distinct patterns in the information-seeking behavior among different groups of cancer patients. We found that older patients and those who were less educated were less likely to seek information, which is consistent with a previous study [26] conducted in Mexico. This study focused on health-related information-seeking behaviors and preferences among Mexican patients with cancer, showing that older age was the characteristic most strongly associated with not seeking information [26]. Moreover, similar results have been found in some high-income countries; Smith-McLallen et al [50] found that level of education and age significantly contributed to the prediction of patients’ seeking intentions in Pennsylvania, while they also indicated that older individuals and those who had lower levels of education were less likely to seek out information from sources other than their doctors. It was also found in a study [2] in Montreal that individuals who avoided information tended to be less educated, while active seekers reported higher education. However, a study [7] in Beijing and Hefei found that age and employment were not related to seeking. Moreover, although education showed some significant relationships with seeking in the predictive models considering predisposing and enabling variables, the relationship became nonsignificant when including need variables [7].

### Practical Suggestions

Given the importance of information seeking to cancer care and outcomes, health practitioners should encourage patients’ information seeking behavior and increase their satisfaction. First, education was found to be an important predictor of information seeking, highlighting the importance of narrowing educational gaps together with economic gaps between southwestern and eastern regions in China. This could generally increase patients’ health literacy since education and health literacy often overlap [34]. Moreover, many Chinese individuals hold the fatalistic beliefs about cancer. Practitioners should understand the roles of these beliefs better before attempting interventions to improve the access and availability of optimal treatments. For example, the relationship between cancer beliefs and traditional Chinese medicine should be considered. Furthermore, cancer patients undergoing treatment in the hospital are seen by the same fellow or oncologist, which facilitates a steady and trustable relationship. This relationship can form a close tie, which is a fundamental value in the Chinese collectivism culture, to benefit information provision. For example, during hospital stays, oncologists can provide tailored messages, tell the patients how to find information, and guide them in appraising information sources. In addition, oncologists may also continue to help patients in their information seeking by using email and social networking sites based on the close relationships.

### Limitations

There are some limitations in our study. For example, this cross-sectional survey was not equipped to explore the causality between variables. Furthermore, this study only investigated several possible factors influencing beliefs and information seeking. Further research is needed to follow a theoretical framework and explore more factors. This survey was conducted in two hospitals in southwest China, and thus may not be generalizable outside of a similar context. Finally, since the participants of all types of cancer were randomly sampled, the results could be biased by the participants with the majority type of cancer and should be interpreted with caution.

### Conclusions

This study explored the information-seeking patterns of cancer patients in southwest China. It was found that many Chinese people endorsed cancer fatalism. These pessimistic beliefs about the potential to prevent and to cure cancer are correlated with cancer-related information seeking, while self-efficacy confidence in finding needed cancer information was a significant predictor of seeking out information. Moreover, this study demonstrated that information seekers and nonseekers differ demographically. These findings reflect a sense of urgency for cancer information dissemination in low- to middle-income regions such as southwest China.

## Appendix

Multimedia Appendix 1 Questionnaire_English version.

Multimedia Appendix 2 The results of collinearity diagnosis.

Multimedia Appendix 3 Results of two linear regressions.

## Abbreviations

ANOVA: analysis of variance
GDP: gross domestic product
HINTS: Health Information National Trends Survey
RMB: Renminbi
