# Supplementary material for: Beliefs and Information Seeking in Patients With Cancer in Southwest China: Survey Study
Source: JMIR Cancer. 2020 Aug 21;6(2):e16138. doi: 10.2196/16138 (PMC7474411; doi:10.2196/16138)
Supplement: Multimedia Appendix 1 [file cancer_v6i2e16138_app1.docx]

# Questionnaire

A: Looking for health information

A1. Have you ever looked for information about health or medical topics from any source?

1. Yes

2. No (If not, go to B.)

A2. The most recent time you looked for information about cancer, where did you go first?

1. Books 2. Cancer organizations 3. Internet 4. Libraries 5. Family members 6. Friends or colleagues 7. Magazines 8. Newspapers 9. Doctors or health care providers 10. Telephone health line

A3. In general, how much would you trust information about cancer from each of the following? (Five represents totally trust while one represents totally distrust.)

|  | 1 | 2 | 3 | 4 | 5 |
| --- | --- | --- | --- | --- | --- |
| a. doctors |  |  |  |  |  |
| b. family or friends |  |  |  |  |  |
| c. newspapers or magazines |  |  |  |  |  |
| d. radio |  |  |  |  |  |
| e. internet |  |  |  |  |  |
| f. television |  |  |  |  |  |
| g. government health agencies |  |  |  |  |  |
| h. charitable organizations |  |  |  |  |  |
| i. religious organizations |  |  |  |  |  |

A4. Based on the results of your most recent search for information about cancer, how much do you agree or disagree with each of the following statements? (Five represents strongly agree while one represents strongly disagree.)

|  | 1 | 2 | 3 | 4 | 5 |
| --- | --- | --- | --- | --- | --- |
| It took a lot of effort to get the information you needed |  |  |  |  |  |
| It took a lot of effort to get the information you needed |  |  |  |  |  |
| You were not interested about the information you found |  |  |  |  |  |
| The information you found was hard to understand |  |  |  |  |  |

B: Using the internet to find information

B1. Do you ever go on-line to access the Internet or World Wide Web, or to send and receive e-mail?

1. Yes

2. no (If not, go to B4.)

B2. When you use the Internet, do you access it through the following ways? (Multiple choice)

1. Broadband or telephone line

2. Cellular network (e.g., phone)

3. Wireless network (Wi-Fi)

B3. In the past 12 months, how often do you used a computer, smartphone, or other electronic means to do any of the following?

|  | never | sometimes | often | always | usually |
| --- | --- | --- | --- | --- | --- |
| a. Looked for health or medical information |  |  |  |  |  |
| b. Looked for a health care provider |  |  |  |  |  |
| c. Bought medicine or vitamins online |  |  |  |  |  |
| d. Filled out forms or paperwork related to your health care |  |  |  |  |  |
| e. Used e-mail or the Internet to communicate with a doctor or a doctor’s office |  |  |  |  |  |
| f. Track health care charges and costs |  |  |  |  |  |

B4. Have you sent or received a text message from a doctor or other health care professional within the last 12 months?

1. Yes

2. No

3. Don’t know

C: Cancer belief and self-belief

C1. How much do you agree or disagree with each of the following statements? (Five represents strongly agree while one represents strongly disagree.)

|  | 1 | 2 | 3 | 4 | 5 |
| --- | --- | --- | --- | --- | --- |
| a. It seems like everything causes cancer |  |  |  |  |  |
| b. There’s not much you can do to lower your chances of getting cancer |  |  |  |  |  |
| c. There are so many different recommendations about preventing cancer, it's hard to know which ones to follow |  |  |  |  |  |
| d. In adults, cancer is more common than heart disease |  |  |  |  |  |
| e. When I think about cancer, I automatically think about death |  |  |  |  |  |

C2. How much do you agree with the following statement? (Five represents strongly agree while one represents strongly disagree.)

|  | 1 | 2 | 3 | 4 | 5 |
| --- | --- | --- | --- | --- | --- |
| You did not feel confident that you could get advice or information about cancer if you needed it |  |  |  |  |  |

C3. Have any of your family members ever had cancer?

1. Yes

2. No

3. Don’t know

D: You and your household

D1. What is your age?

D2. How many children under the age of 18 live in your household?

D3. What is your current occupational status?

1. Employed

2. Unemployed

3. Student

4. Retired

5. Homemaker

6. Farmer

D4. What is your marital status?

1. Single, never married

2. Married

3. Widowed

4. Divorced

D5. What is the highest grade or level of schooling you completed?

1. <Primary school

2. Junior school

3. High school

4. College

5. Bachelor’s degree, or higher

D6. Do you currently rent or own your home?

1. Own

2. Rent

3. Occupied without paying monetary rent

D7. What is the annual income of your household, meaning the total pre-tax income from all sources earned in the past year?

1. <10,000 RMB

2. 10,000-50,000RMB;

3. 50,000-100,000RMB

4. >100,000RMB
