# Supplementary material for: Beliefs and Information Seeking in Patients With Cancer in Southwest China: Survey Study
Source: JMIR Cancer. 2020 Aug 21;6(2):e16138. doi: 10.2196/16138 (PMC7474411; doi:10.2196/16138)
Supplement: Multimedia Appendix 2 [file cancer_v6i2e16138_app2.docx]

Table 1 the results of collinearity diagnostics ^a^

| Model | Dimension | Eigenvalue | Condition index | Variance ratio | | | | | | | |
| --- | --- | --- | --- | --- | --- | --- | --- | --- | --- | --- | --- |
|  |  |  |  | constant | age | D2marital=1.0 | D2marital=2.0 | D2marital=4.0 | A3e_efficacy | D3education | D5income |
| 1 | 1 | 5.381 | 1.000 | .00 | .00 | .00 | .00 | .00 | .00 | .00 | .01 |
|  | 2 | 1.054 | 2.259 | .00 | .00 | .24 | .01 | .03 | .00 | .00 | .00 |
|  | 3 | 1.002 | 2.318 | .00 | .00 | .01 | .01 | .40 | .00 | .00 | .00 |
|  | 4 | .196 | 5.245 | .01 | .02 | .04 | .03 | .01 | .01 | .01 | .81 |
|  | 5 | .163 | 5.744 | .00 | .16 | .03 | .06 | .05 | .06 | .15 | .06 |
|  | 6 | .119 | 6.716 | .00 | .03 | .22 | .34 | .10 | .03 | .32 | .09 |
|  | 7 | .073 | 8.561 | .00 | .01 | .06 | .07 | .04 | .67 | .52 | .03 |
|  | 8 | .012 | 21.402 | .99 | .78 | .41 | .48 | .37 | .23 | .00 | .00 |
| a. dependent variable：cancerbelief (cancer fatalism) | | | | | | | | | | | |

Table 2 linear regression analysis for collinearity diagnostics ^a^

| Model | | Unstandardized coefficients | | Standardized coefficients | t | Sig. | Collinearity diagnostics | |
| --- | --- | --- | --- | --- | --- | --- | --- | --- |
|  |  | B | se | Beta |  |  | tolerance | VIF |
| 1 | Constant | 3.596 | .167 |  | 21.592 | .000 |  |  |
|  | Age | .005 | .002 | .181 | 3.223 | .001 | .531 | 1.882 |
|  | D2marital=1.0 | .014 | .126 | .008 | .113 | .911 | .343 | 2.914 |
|  | D2marital=2.0 | .003 | .080 | .003 | .040 | .968 | .353 | 2.830 |
|  | D2marital=4.0 | .046 | .110 | .024 | .417 | .677 | .503 | 1.986 |
|  | A3e_efficacy | .008 | .024 | .016 | .317 | .751 | .641 | 1.560 |
|  | D3education | -.276 | .020 | -.693 | -13.651 | .000 | .646 | 1.547 |
|  | D5income | .032 | .020 | .070 | 1.607 | .109 | .875 | 1.143 |
| a. dependent variable：cancerbelief (cancer fatalism) | | | | | | | | |
