# Supplementary material for: Beliefs and Information Seeking in Patients With Cancer in Southwest China: Survey Study
Source: JMIR Cancer. 2020 Aug 21;6(2):e16138. doi: 10.2196/16138 (PMC7474411; doi:10.2196/16138)
Supplement: Multimedia Appendix 3 [file cancer_v6i2e16138_app3.docx]

Table 1 Linear regression analysis between demographics and self-efficacy ^a^

| model | | Unstandardized coefficients | | Standardized coefficients | t | Sig. |
| --- | --- | --- | --- | --- | --- | --- |
|  |  | B | se | Beta |  |  |
| 1 | Constant | 3.517 | .249 |  | 14.098 | .000 |
|  | Age | -.019 | .004 | -.294 | -4.587 | .000 |
|  | D2marital=1.0 | .255 | .221 | .066 | 1.157 | .248 |
|  | D2marital=3.0 | .205 | .203 | .056 | 1.011 | .313 |
|  | D2mariatl=4.0 | -.220 | .202 | -.054 | -1.090 | .277 |
|  | D3education=2.0 | .035 | .156 | .014 | .227 | .821 |
|  | D3education=3.0 | .400 | .160 | .152 | 2.494 | .013 |
|  | D3education=4.0 | .814 | .189 | .266 | 4.316 | .000 |
|  | D3education=5.0 | 1.308 | .212 | .380 | 6.160 | .000 |
|  | D5income=2.0 | -.210 | .146 | -.076 | -1.441 | .151 |
|  | D5income=3.0 | -.180 | .160 | -.057 | -1.125 | .261 |
|  | D5income=4.0 | .187 | .168 | .062 | 1.109 | .268 |
| a. dependent variable：A3e_efficacy | | | | | | |

Table 2 Linear regression analysis between demographics and cancer fatalism ^a^

| model | | Unstandardized coefficients | | Standardized coefficients | t | Sig. |
| --- | --- | --- | --- | --- | --- | --- |
|  |  | B | se | Beta |  |  |
| 1 | Constant | 3.414 | .101 |  | 33.950 | .000 |
|  | Age | .005 | .002 | .167 | 3.017 | .003 |
|  | D2marital=1.0 | -.008 | .089 | -.004 | -.086 | .931 |
|  | D2marital=3.0 | -.018 | .082 | -.010 | -.216 | .829 |
|  | D2marital=4.0 | .025 | .081 | .013 | .303 | .762 |
|  | D3education=2.0 | -.330 | .063 | -.277 | -5.242 | .000 |
|  | D3education=3.0 | -.584 | .065 | -.476 | -9.030 | .000 |
|  | D3education=4.0 | -.834 | .076 | -.585 | -10.981 | .000 |
|  | D3education=5.0 | -1.070 | .086 | -.666 | -12.496 | .000 |
|  | D5income=2.0 | .058 | .059 | .045 | .992 | .322 |
|  | D5income=3.0 | .136 | .064 | .093 | 2.118 | .035 |
|  | D5income=4.0 | .048 | .068 | .034 | .713 | .476 |
| a. dependent variable：cancerbelief (cancer fatalism) | | | | | | |
